# Shexiang Baoxin Pills for Coronary Heart Disease in Animal Models: Preclinical Evidence and Promoting Angiogenesis Mechanism

**DOI:** 10.3389/fphar.2017.00404

**Published:** 2017-06-28

**Authors:** Ke-Jian Zhang, Jia-Zhen Zhu, Xiao-Yi Bao, Qun Zheng, Guo-qing Zheng, Yan Wang

**Affiliations:** Department of Cardiology, The Second Affiliated Hospital and Yuying Children's Hospital of Wenzhou Medical UniversityWenzhou, China

**Keywords:** angiogenesis, myocardial infarction, cardiovascular polypill, traditional Chinese medicine, TCM compound formula

## Abstract

Shexiang Baoxin Pill (SBP) originated from a classical TCM Fufang Suhexiang Pill for chest pain with dyspnea in the Southern Song Dynasty (1107–110 AD). Here, we aimed to evaluate preclinical evidence and possible mechanism of SBP for experimental coronary heart disease (CHD). Studies of SBP in animal models with CHD were identified from 6 databases until April 2016. Study quality for each included article was evaluated according to the CAMARADES 10-item checklist. Outcome measures were myocardial infarction area, vascular endothelial growth factor (VEGF) and microvessel count (MVC). All the data were analyzed by using RevMan 5.1 software. As a consequence, 25 studies with 439 animals were identified. The quality score of studies ranged from 2 to 5, with the median of 3.6. Meta-analysis of seven studies showed more significant effects of SBP on the reduction of the myocardial infarction area than the control (*P* < 0.01). Meta-analysis of eight studies showed significant effects of SBP for increasing VEGF expression compared with the control (*P* < 0.01). Meta-analysis of 10 studies indicated that SBP significantly improved MVC compared with the control (*P* < 0.01). In conclusion, these findings preliminarily demonstrated that SBP can reduce myocardial infarction area, exerting cardioprotective function largely through promoting angiogenesis.

## Introduction

Traditional Chinese Medicine (TCM) is one of the oldest continuous healing systems in healthcare with the history of over 2,000 years (Tang et al., 2008) and has formed a uniquely holistic medical system to diagnose and cure illness. TCM compound formula (Fufang), a pharmaceutical therapeutic modality of TCM, referred to a combination of several Chinese materia medica derived from multiple plants, mineral, or occasionally animal sources based on the principle of Jun-Chen-Zuo-Shi (also known as emperor-minister-assistant-courier), first recorded by *Huangdi Neijing* (*Huangdi's Internal Classic*; Fan et al., 2006). A combinatorial TCM Fufang against cardiovascular disease carries similarities to the cardiovascular polypill used in conventional western medicine (Xiang et al., 2012). The cardiovascular polypill was defined as an innovative, simple and cost-effective public health strategy, known as a multidrug combination therapy, for combating the cardiovascular disease at a global scale (Castellano et al., 2015).

Shexiang Baoxin Pill (SBP) is a featured TCM Fufang for treating cardiovascular disease, which originates from a classical TCM Fufang Suhexiang Pill for chest pain with dyspnea recorded in *Taiping Huimin Heji Jufang* (*Prescriptions from the Great Peace Imperial Grace Pharmacy*) in the Southern Song Dynasty (1107–110 AD). The modern patent prescription of SBP was developed by Dai (2000) group in 1981, which comprises seven Chinese materia medicas as follows: (A) artificial Moschus; musk; the dried preputial secretion of Moschus berezovskii Flerov or Moschus sifanicus Przewalski or Moschus moschiferus Linnaeus; (B) Radix Ginseng; ginseng; the dried root of Panax ginseng C. A. Mey.; (C) Cortex Cinnamomi; cassia bark; the dried bark of Cinnamomum cassia Presl; (D) Venenum Bufonis; toad venom; the dried secretion of Bufo bufo gargarizans Cantor or Bufo melanostictus Schneider; (E) Styrax; storax; the Processed and refined balsam obtained from the wood and inner bark of Liquidambar orientalis Mill; (F) artificial Calculus Bovis; bezoar; the dried gall-stone of Bos taurus domesticus Gmelin; (G) Borneolum Syntheticum; borneol; artificial synthetic product of C_10_H_18_O. Currently, SBP is widely used for the treatment of cardiovascular disease in China and some randomized controlled trials (RCTs) provided evidence to support for the clinical use of SBP for coronary heart disease (CHD; Zhou et al., 2016). The therapeutic mechanisms of the SBP have progressed substantially because many studies claimed that SBP can facilitate the therapeutic angiogenesis. In addition, a systematic review is a literature review to address a specific research question by seeking to identify, select, appraise, and synthesize all available research evidence relevant to that question. Using systematic review as tool to synthesize animal studies can independently evaluate the strength of the preclinical evidence and clarify the emerging mechanisms. Thus, the aim of this systematic review was to assess current preclinical evidence and possible mechanism of SBP for CHD.

## Methods

### Search strategies

The following databases were electronically searched from PubMed, Web of Science, EMBASE, Chinese National Knowledge Infrastructure (CNKI), VIP information database and Wanfang data Information Site from inception to April 2016. The search terms were as follows: “Shexiang Baoxin OR She Xiang Bao Xin” AND “myocardial infarction OR myocardial ischemia” AND “vascular endothelial growth factor OR VEGF OR microvessel count OR MVC.” All searches were limited to studies on animals. We also manually searched published abstracts of scientific meetings and asked senior authors of identified publications for references of related studies.

### Inclusion criteria

To prevent bias, pre-specified inclusion criteria were as follows: (1) experimental CHD model was induced by temporary or permanent myocardial infarction (MI); (2) treatment group received the SBP intervention merely; (3) a control group received vehicle or no treatment; (4) the outcome measures were myocardial infarction area and/or VEGF, and/or MVC; (5) regardless of language, blinding, or publication status.

### Exclusion criteria

Prespecified exclusion criteria met any one of the following conditions: (1) treatment group without SBP or combined use of any other agents; (2) non-CHD animal model; (3) no control group; (4) duplicate publications; (5) reviews, case reports, abstracts, letters, comments, study protocol, editorials, and clinical guidelines.

### Data collection

Two authors independently screened the abstracts, and the included manuscripts were approved by corresponding author. The information extracted from the complete manuscripts of the qualified studies was as follows: (1) the first author's name and publication year, model of CHD (transient or permanent, ligation or drug); (2) the features of animals such as animal number, species, sex, weight, age, and any comorbidity; (3) the information of treatment used in experimental group including the types of SBP, dose, method of administration, and duration of treatment; (4) outcome measures, especially the primary outcomes containing myocardial infarction area and/or VEGF, and/or MVC. If outcomes were performed at different time points, only the final test was included. If the experimental group of animals received various doses of the drug therapy, only the data of highest dose of the drug was included. If published data were incomplete, we contacted authors to obtain further information. For each comparison, we extracted data of mean value and standard deviation from each experimental and control group of every study.

### Quality assessment

We evaluated the methodological quality of the included studies by using the Collaborative Approach to Meta-Analysis and Review of Animal Data from Experimental Studies (CAMARADES) with 10-item quality checklist (MacLeod et al., 2004). One point was awarded for each of (1) publication in a peer-reviewed journal; (2) statement of temperature control; (3) random allocation to groups; (4) allocation concealment; (5) blinded assessment of outcome; (6) use of anesthetic without significant internal protection of blood vessel; (7) appropriate animal model (aged, healthy, diabetic, or hypertensive); (8) sample size calculation; (9) compliance with animal welfare regulations; (10) statement of potential conflict of interests. Two authors independently assessed study quality and any disagreements were solved through discussion or consultation with corresponding author.

### Statistical analysis

All values of myocardial infarction area, VEGF and MVC were considered as continuous data, and then an estimate of the combined effect sizes utilizing standard mean difference (SMD) with the random effects model was given. In the present meta-analysis, the results using the random effects model were presented because heterogeneity between multistudies has to be taken into account. *I*^2^ statistic was used to assess heterogeneity. If significant heterogeneity with *I*^2^-values over 50% was identified, then additional subgroup and/or sensitivity analyses were performed. Probability values 0.05 were considered significant. All analyses were performed with Revman version 5.1 provided by the Cochrane Collaboration.

## Results

### Study inclusion

**Figure 1:**
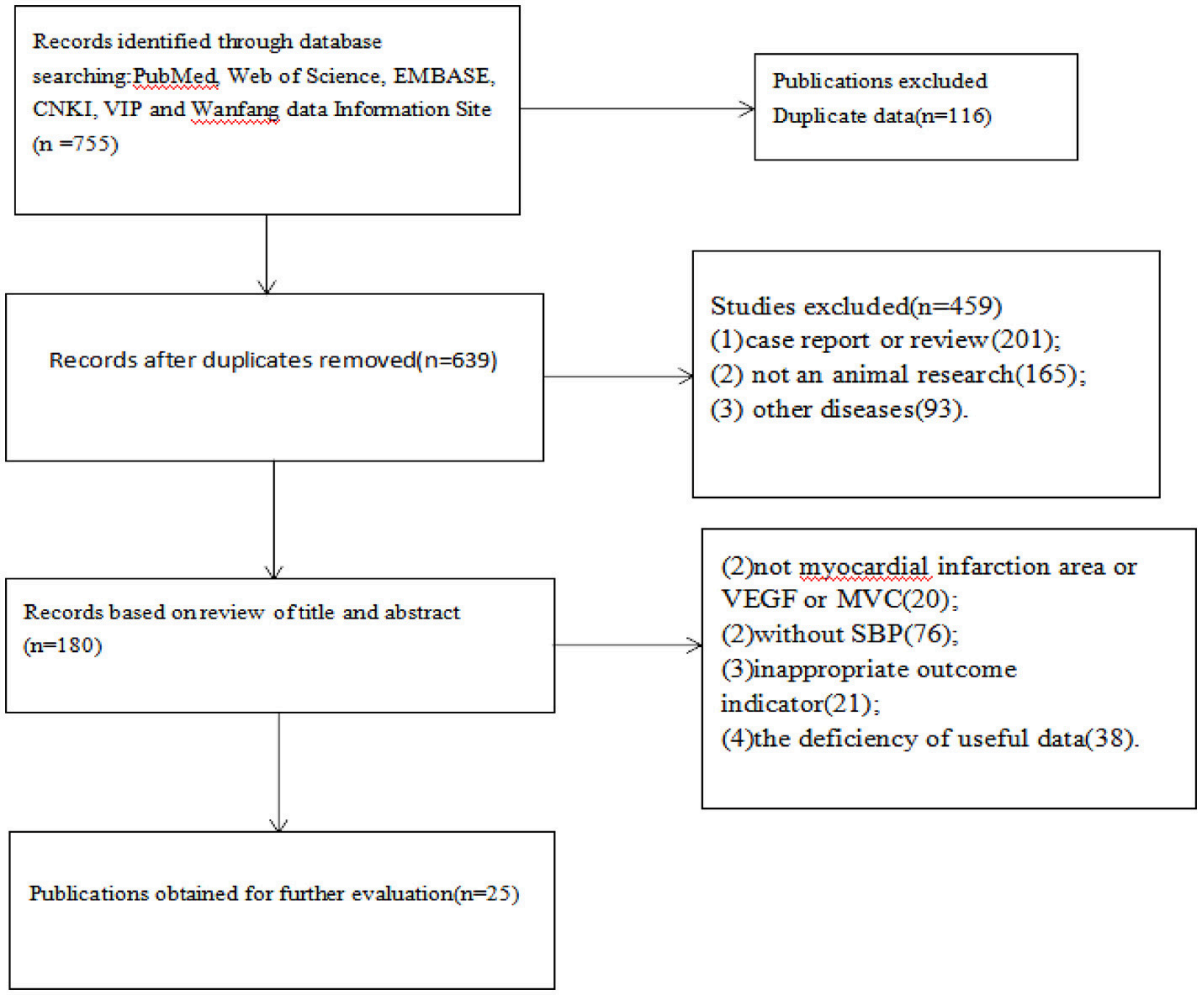
Flow diagram.

We identified 755 potentially relevant articles from six databases. After removal of duplicates, 639 records remained. After going through the titles and the abstracts, we excluded 459 papers with at least one of following reasons: (1) case report or review; (2) not an animal research; and (3) other diseases. Reading the full text of the 180 articles remaining which reported the efficacy of SBP in animal models of myocardial infarction OR myocardial ischemia, 20 studies were excluded because the outcome measure was not myocardial infarction area or VEGF or MVC; 76 studies were excluded without SBP; 21 were excluded because of inappropriate outcome indicators; 38 studies were removed due to the deficiency of useful data. Ultimately, 25 eligible studies were identified (Figure 1; Wang et al., 2002, 2004, 2006, 2007, 2014; Han et al., 2006, 2007; Huang and Huang, 2006; Ling et al., 2007; Chen and Yuan, 2008; Li et al., 2008; Shen and Fan, 2008; Tian and Wang, 2008; Wang and Fan, 2008; Xie and Chen, 2008; Yang et al., 2010; Zhang Q. Y. et al., 2011; Zhang S. J. et al., 2011; Guo et al., 2013; Huang et al., 2013, 2014; Luan et al., 2013, 2014; Zang et al., 2014; Yuan et al., 2015).

### Study characteristics

A total of 439 subjects were included in the 25 studies, of whom 228 were in the experimental group and 211 were in the control group. Twenty-one studies were published in Chinese between 2002 and 2015, and seven studies have not been published as Mphil or PhD thesis. Nine studies (Wang et al., 2004, 2014; Wang and Fan, 2008; Xie and Chen, 2008; Zhang Q. Y. et al., 2011; Guo et al., 2013; Huang et al., 2013, 2014; Zang et al., 2014) used male/female Sprague-Dawley rat models; 14 studies (Wang et al., 2004, 2006, 2007; Han et al., 2006, 2007; Huang and Huang, 2006; Chen and Yuan, 2008; Li et al., 2008; Tian and Wang, 2008; Yang et al., 2010; Zhang S. J. et al., 2011; Luan et al., 2013, 2014; Yuan et al., 2015) used male Wistar rats; one study (Shen and Fan, 2008) used male New-Zealand rabbits; one study (Ling et al., 2007) used female Japan-Sino hybridization white rabbits. All the studies used permanent CHD models which were divided into ligation (Wang et al., 2002, 2004, 2006, 2007, 2014; Han et al., 2006, 2007; Huang and Huang, 2006; Ling et al., 2007; Chen and Yuan, 2008; Li et al., 2008; Tian and Wang, 2008; Wang and Fan, 2008; Xie and Chen, 2008; Yang et al., 2010; Zhang Q. Y. et al., 2011; Zhang S. J. et al., 2011; Guo et al., 2013; Huang et al., 2013, 2014; Luan et al., 2013, 2014; Zang et al., 2014; Yuan et al., 2015) and embolism with the obstructive glue (Shen and Fan, 2008) of the left descending coronary artery. The dosage, duration and initial administration time in the studies aren't all the same. The dosage administrated is various as follows: 0.03 g/kg in 11 studies (Wang et al., 2002, 2006, 2007, 2014; Han et al., 2006, 2007; Yang et al., 2010; Zhang Q. Y. et al., 2011; Zhang S. J. et al., 2011; Guo et al., 2013; Yuan et al., 2015); 0.08 g/kg in four studies (Huang and Huang, 2006; Xie and Chen, 2008; Huang et al., 2013, 2014); 0.1 g/kg in two studies (Wang and Fan, 2008; Zang et al., 2014); 0.05 g/kg in two studies (Wang et al., 2004; Ling et al., 2007); 0.0142 g/kg in two studies (Luan et al., 2013, 2014); the remaining four studies different from each other. The durations of administration time are diverse, including 6 weeks in 12 studies (Wang et al., 2002, 2006, 2007; Han et al., 2006, 2007; Shen and Fan, 2008; Xie and Chen, 2008; Yang et al., 2010; Zhang S. J. et al., 2011; Luan et al., 2013, 2014; Huang et al., 2014), 2 weeks in five studies (Huang and Huang, 2006; Ling et al., 2007; Chen and Yuan, 2008; Guo et al., 2013; Wang et al., 2014), 4 weeks in three studies (Tian and Wang, 2008; Huang et al., 2013; Yuan et al., 2015), 8 weeks in two studies (Wang et al., 2004; Wang and Fan, 2008), 1 week in two studies (Li et al., 2008; Zhang Q. Y. et al., 2011) and 3 weeks in one study (Zang et al., 2014). The initial time to be given SBP is as follows: 1 day after myocardial infarction in 23 studies (Wang et al., 2002, 2004, 2006, 2007, 2014; Han et al., 2006, 2007; Huang and Huang, 2006; Ling et al., 2007; Chen and Yuan, 2008; Li et al., 2008; Tian and Wang, 2008; Wang and Fan, 2008; Yang et al., 2010; Zhang Q. Y. et al., 2011; Zhang S. J. et al., 2011; Guo et al., 2013; Huang et al., 2013, 2014; Luan et al., 2013, 2014; Zang et al., 2014; Yuan et al., 2015) and 1 week after myocardial infarction in two studies (Shen and Fan, 2008; Xie and Chen, 2008).

Eight studies (Wang et al., 2002, 2004; Li et al., 2008; Tian and Wang, 2008; Wang and Fan, 2008; Xie and Chen, 2008; Huang et al., 2013, 2014) reported myocardial infarction area, 12 studies (Wang et al., 2002, 2006, 2014; Huang and Huang, 2006; Ling et al., 2007; Tian and Wang, 2008; Xie and Chen, 2008; Yang et al., 2010; Zhang Q. Y. et al., 2011; Guo et al., 2013; Huang et al., 2014; Zang et al., 2014) VEGF protein, five studies (Chen and Yuan, 2008; Li et al., 2008; Shen and Fan, 2008; Zhang S. J. et al., 2011; Luan et al., 2013) VEGF mRNA, 12 studies (Han et al., 2006, 2007; Wang et al., 2006, 2007; Chen and Yuan, 2008; Yang et al., 2010; Zhang Q. Y. et al., 2011; Zhang S. J. et al., 2011; Huang et al., 2013; Luan et al., 2014; Zang et al., 2014; Yuan et al., 2015) MVC. The characteristics of the 25 included studies were summarized in detail in Table 1.

**Table 1:** Characteristics of the included studies.

**Study (years)**	**Species (Sex, experimental/ control group)**	**Weight**	**Model method**	**Random method**	**Anesthetic**	**Experimental group**	**Control group**	**Outcome**	**Intergroup differences**
Xie and Chen, 2008	Male, Sprague Dawley rats (12/12)	180–220 g	Occlusion	Not mentioned	Pentobarbital sodium	7 days after surgery SBP, 6 weeks 0.08 g/kg	7 days after surgery NS, 6 weeks	Myocardial infarction area MVD VEGF Flt-1 protein	*P* < 0.01 *P* < 0.01 *P* < 0.01 *P* < 0.01
Wang and Fan, 2008	Male, Sprague Dawley rats(11/12)	300–340 g	Occlusion	Not mentioned	Pentobarbital sodium	After surgery, SBP, 8 weeks 0.1 g/Kg	After surgery NS, 8 weeks	MVD Myocardial infarction area LVW/BW Angiotensin II Angiotensin II mRNA Collagen I/III	*P* < 0.05 *P* < 0.05 *P* < 0.05 *P* < 0.05 *P* < 0.05 *P* < 0.05
Chen and Yuan, 2008	Male, Wistar rats (6/6)	240–260 g	Occlusion	Not mentioned	Chloral hydrate	1 day after surgery SBP, 2 weeks 4 ml	1 day after Surgery NS, 2 weeks	MVC bFGF VEGFmRNA bFGFmRNA	*P* < 0.05 *P* < 0.05 *P* < 0.05 *P* < 0.05
Shen and Fan, 2008	Male, New Zealand rabbits(8/8)	2,700–3,000 g	Embolism with the obstructve Glue	Not mentioned	sumianxin	1 weeks Before surgery SBP 6 weeks After operation 45 mg	1 weeks Before surgery 6 weeks After operation NS	Blood fat Heart function Marginal zone aorta vessel wall MVD Myocardial infarction HIF-la mRNA myocardial infarction VEGF mRNA myocardial infarction VEGF2mRNA HIF⇀la VEGFR2	– *P* < 0.01 *P* < 0.01 *P* < 0.01 – *P* < 0.05 *P* < 0.01 – *P* < 0.01
Wang et al., 2007	Male, Wistar rats (8/8)	180–220 g	Occlusion	Not mentioned	Pentobarbital sodium	After surgery SBP 6 weeks 0.03 g/kg	After surgery NS,6 weeks	MVC bFGF PDGF-B IGF-1	*P* < 0.05 *P* < 0.05 *P* < 0.05 *P* < 0.05
Li et al., 2008	Male, Wistar rats (7/6)	180–220 g	Occlusion	Not mentioned	Pentobarbital sodium	1 day after surgery SBP, 1 week 0.0122 g/kg	1 day after Surgery Distilled water,1 week	Myocardial infarction area HGFmRNA VEGFmRNA	*P* < 0.05 *P* < 0.05 *P* < 0.05
Tian and Wang, 2008	Male, Wistar rats(6/6)	220–260 g	Occlusion	Random number table	Chloral hydrate	1 day after surgery SBP, 4 weeks 2 mg/kg	1 day after Surgery Distilled water 4 weeks	Left ventricular ejection fraction Blood serum VEGF content Myocardial infraction area MVD VEGF	*P* < 0.05 *P* < 0.01 *P* < 0.05 *P* < 0.01 *P* < 0.01
Yuan et al., 2015	Male, Wistar rats(20/20)	180–220 g	Occlusion	Not mentioned	Pentobarbital sodium	1 day after surgery SBP, 4 weeks 0.03 g/kg	1 day after surgery NS, 4 weeks	LVEF, LVFS, LVED MVC	*P* < 0.05, *P* > 0.05, *P* < 0.05 *P* < 0.05
Ling et al., 2007	Female, JaPan-sino hybridization white rabbits (8/5)	2,000–2,600 g	Occlusion	Not mentioned	Chloral hydrate	After surgery SBP, 2 weeks 0.05 g/kg	After surgery NS, 2 weeks	bFGF VEGF	*P* > 0.05 *P* > 0.05
Huang et al., 2013	Female, *SD* rats (5/5)	210–250 g	Occlusion	Not mentioned	Chloral hydrate	After surgery SBP, 4 weeks 0.08 g/kg	After Surgery NS, 4 weeks	Myocardial infarction area MVC MVD	*P* < 0.01 *P* < 0.05 *P* < 0.05
Han et al., 2006	Male, Wistar rats(9/7)	210–250 g	Occlusion	Not mentioned	Pentobarbital sodium	1 day after surgery SBP, 6 weeks 0.0 3 g/kg	1 day after surgery NS, 6 weeks	MVC VEGF-B	*P* < 0.05 *P* < 0.05
Wang et al., 2006	Male, Wistar rats (8/8)	180–220 g	Occlusion	Not mentioned	Pentobarbital sodium	1 day after surgery SBP, 6 weeks 0.03 g/kg	1 day after surgery NS, 6 weeks	MVC VEGF bFGF PDGF-β IGF-1	*P* < 0.05 *P* < 0.05 *P* < 0.05 *P* < 0.05 *P* < 0.05
Huang and Huang, 2006	Male, Wistar rats(10/7)	300–350 g	Occlusion	Weight random number table	Ether	1 day after surgery SBP 2 weeks 0.08 g/kg	1 day after surgery Drinking water,2 weeks	SOD MDA NO VEGF	*P* < 0.01 *P* < 0.01 *P* < 0.01 *P* < 0.01
Luan et al., 2013	Male, Wistar rats(12/10)	220–260 g	Occlusion	Not mentioned	Ether	1 day after surgery SBP 6 weeks 0.0142 g/kg	1 day after surgery sodium carboxymethylcellulose 6 weeks	VEGF bFGF VEGF mRNA bFGF mRNA	*P* < 0.001 *P* < 0.05 *P* < 0.001 *P* < 0.01
Luan et al., 2014	Male, Wistar rats(12/10)	220–260 g	Occlusion	Not mentioned	Ether	1 day after surgery SBP 6 weeks 0.0142 g/kg	1 day after surgery sodium carboxymethylcellulose 6 weeks	MVC Myocardial infarction area	*P* < 0.01 *P* < 0.01
Yang et al., 2010	Male, Wistar rats (8/7)	220–280 g	Occlusion	Not mentioned	Chloral hydrate	In 1 day after surgery SBP 6 weeks 0.03 g/kg	In 1 day after surgery Distilled water 6 weeks	MVC VEGF bFGF	*P* < 0.05 *P* < 0.05 *P* < 0.05
Guo et al., 2013	Male/Female, SD rats (8/8)	240–260 g	Occlusion	Not mentioned	Chloral hydrate	1 day after surgery SBP 2 weeks 0.03 g/kg	1 day after Surgery Distilled water 2 weeks	MVD Notch1 VEGF	*P* < 0.05 *P* < 0.01 *P* < 0.01
Zhang Q. Y. et al., 2011	Male, SD rats (8/8)	150–200 g	Occlusion	Not mentioned	Ethyl carbamate	1 day after surgery SBP 0.03 g/kg 1 week	1 day after Surgery Distilled water, 1 week	MVD VEGF MVC	*P* < 0.05 *P* < 0.05 *P* < 0.05
Wang et al., 2014	Male/Female, SD rats (8/8)	220–280 g	Occlusion	Not mentioned	Chloral hydrate	1 day after surgery SBP 2 weeks 0.03 g/kg	1 day after surgery Distilled water 2 weeks	NO VEGF DLL4	*P* < 0.01 *P* < 0.01 *P* < 0.01
Zhang S. J. et al., 2011	Male, Wistar rats (8/8)	180–220 g	Occlusion	Not mentioned	Pentobarbital sodium	In 1 day after surgery SBP 6 weeks 0.03 g/kg	In 1 day after surgery NS 6 weeks	MVC VEGF mRNA	*P* < 0.05 *P* < 0.05
Huang et al., 2014	Female, SD rats (12/10)	180–220 g	Occlusion	Not mentioned	Pentobarbital sodium	After surgery SBP 6 weeks 0.08 g/kg	After surgery NS 2 ml 6 weeks	HIF VEGF eNOS mRNA myocardial infarction area	*P* < 0.05 *P* < 0.05 *P* < 0.05 *P* < 0.01
Zang et al., 2014	Male, SD rats (5/5)	About 200 g	Occlusion	Not mentioned	Chloral hydrate	After 1 day after surgery SBP 3 weeks 0.1 g/kg	After 1 day after surgery NS 3 weeks	MVC VEGF, bFGF	*P* < 0.01 *P* < 0.01
Han et al., 2007	Male, Wistar rats (9/7)	210–250 g	Occlusion	Not mentioned	Pentobarbital sodium	In 1 day after surgery SBP 6 weeks 0.03 g/kg	In 1 day after Surgery NS 6 weeks	MVD MVC PDGF-B	*P* < 0.05 *P* < 0.05 *P* < 0.05
Wang et al., 2002	Male, Wistar rats (8/8)	300–340 g	Occlusion	Not mentioned	Ketamine hydrochloride	After surgery SBP 6 weeks 0.03 g/kg	After surgery NS 4 ml 6 weeks	Myocardial infarction area VEGF, bFGF MVD	*P* < 0.05 *P* < 0.01 *P* < 0.01
Wang et al., 2004	Male, SD rats (12/12)	300–340 g	Occlusion	Not mentioned	Ketamine hydrochloride	After surgery SBP 8 weeks 0.05 g/kg	After surgery NS 2 ml 8 weeks	Myocardial infarction area MVD	*P* < 0.05 *P* < 0.05

### Study quality

Eighteen studies were publications in a peer reviewed journal. Seventeen studies (Han et al., 2006; Huang and Huang, 2006; Ling et al., 2007; Chen and Yuan, 2008; Li et al., 2008; Shen and Fan, 2008; Tian and Wang, 2008; Xie and Chen, 2008; Yang et al., 2010; Zhang Q. Y. et al., 2011; Guo et al., 2013; Huang et al., 2013, 2014; Luan et al., 2013, 2014; Zang et al., 2014; Yuan et al., 2015) reported the control of temperature. All studies described random allocation to groups, of which two studies (Huang and Huang, 2006; Tian and Wang, 2008) used random number table method. None of the studies used either allocation concealment or blinded assessment of outcome. Chloral hydrate was used as anesthetic in eight studies (Ling et al., 2007; Chen and Yuan, 2008; Tian and Wang, 2008; Yang et al., 2010; Guo et al., 2013; Huang et al., 2013; Wang et al., 2014; Zang et al., 2014); pentobarbital was used in 10 studies (Han et al., 2006, 2007; Wang et al., 2006, 2007; Li et al., 2008; Wang and Fan, 2008; Xie and Chen, 2008; Zhang S. J. et al., 2011; Huang et al., 2014; Yuan et al., 2015); sumianxin (compound preparation, including Xylidinothiazole, Ethylenediamine tetraacetic acid, Dihydroetorphine Hydrochloride, and Haloperidol) was used in one study (Shen and Fan, 2008); ether was used in two studies (Huang and Huang, 2006; Luan et al., 2013, 2014); ketamine hydrochloride was used in two studies (Wang et al., 2002, 2004); ethyl carbamate was used in one study (Wang et al., 2004). No studies described a sample size calculation, and none of studies reported compliance with animal welfare regulations or mentioned a statement of potential conflict of interests. One study (Yuan et al., 2015) chose the healthy rats with the standard II lead ECG normal, one (Guo et al., 2013) chose the healthy adult rats, one (Huang et al., 2014) chose the healthy rats, two (Shen and Fan, 2008; Xie and Chen, 2008) chose the rats high-fat, and the others chose the appropriate animal models but not described the characteristics. The quality score of studies ranges from 2 to 5, and the median was 3.6. The methodological quality of each study was summarized in Table 2.

**Table 2:** Risk of bias of the included studies.

**Study**	**1**	**2**	**3**	**4**	**5**	**6**	**7**	**8**	**9**	**10**	**Total**
Xie and Chen, 2008		√	√			√	√				4
Wang and Fan, 2008			√			√					2
Chen and Yuan, 2008		√	√			√					3
Shen and Fan, 2008		√	√			√	√				4
Wang et al., 2007	√		√			√					3
Li et al., 2008	√	√	√			√					4
Tian and Wang, 2008		√	√			√					3
Yuan et al., 2015	√	√	√			√	√				5
Ling et al., 2007		√	√			√					3
Huang et al., 2013	√	√	√			√					4
Han et al., 2006	√	√	√			√					4
Wang et al., 2006	√		√			√					3
Huang and Huang, 2006	√	√	√			√					4
Luan et al., 2013	√	√	√			√					4
Luan et al., 2014	√	√	√			√					4
Yang et al., 2010	√	√	√			√					4
Guo et al., 2013		√	√			√	√				4
Zhang Q. Y. et al., 2011	√	√	√			√					4
Wang et al., 2014	√		√			√					3
Zhang S. J. et al., 2011	√		√			√					3
Huang et al., 2014	√	√	√			√	√				5
Zang et al., 2014	√	√	√			√					4
Han et al., 2007	√		√			√					3
Wang et al., 2002	√		√			√					3
Wang et al., 2004	√		√			√					3

*(1) Publication in a peer-reviewed journal; (2) Statement of temperature control; (3) Random allocation to groups; (4) Allocation concealment; (5) blinded assessment of outcome; (6) Use of anesthetic without significant Internal blood vessel; (7) Appropriate animal model (aged, diabetic, or hypertensive); (8) Sample size calculation; (9) Compliance with animal welfare regulations; (10) Statement of potential conflict of interests*.

### Effectiveness

#### Myocardial infarction area

**Figure 2:**
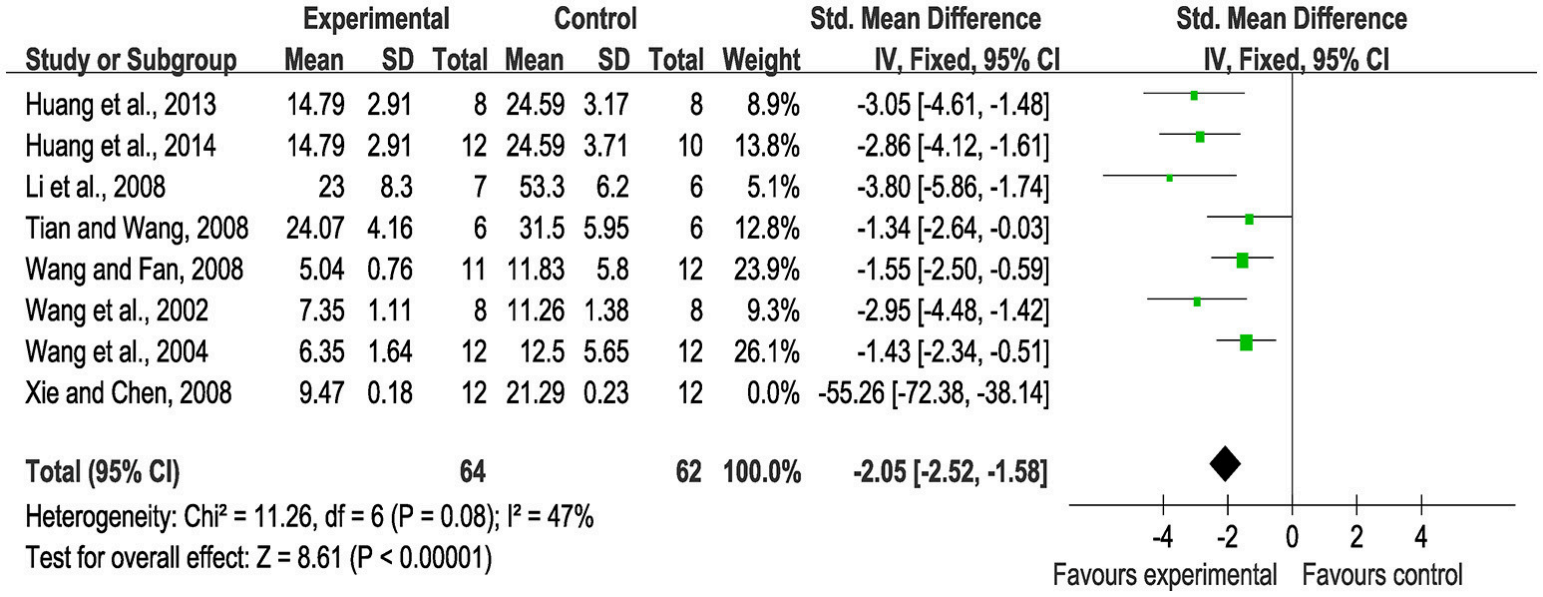
The forest plot: effects of Shexiang Baoxin Pills for reducing myocardial infarction area.

Meta-analysis of eight studies (Wang et al., 2002, 2004; Li et al., 2008; Tian and Wang, 2008; Wang and Fan, 2008; Xie and Chen, 2008; Huang et al., 2013, 2014) showed significant effects of SBP for reducing myocardial infarction area compared with control (*n* = 150, SMD: −2.09, 95% CI: −2.56~−1.62, *P* < 0.00001; heterogeneity χ^2^ = 48.35, *P* < 0.00001, *I*^2^ = 86%). A sensitivity analysis was conducted by sequentially excluding one study. After excluding the study (Xie and Chen, 2008) because the rats in this study administrated SBP 1 week post-model established, meta-analysis of seven studies (Wang et al., 2002, 2004; Li et al., 2008; Tian and Wang, 2008; Wang and Fan, 2008; Huang et al., 2013, 2014) demonstrated more significant effects of SBP on the reduction of the myocardial infarction area than the control (*n* = 126, SMD: −2.05, 95% CI: −2.52~−1.58, *P* < 0.00001; heterogeneity χ^2^ = 11.26, *P* = 0.08, *I*^2^ = 47%), Figure 2.

#### VEGF

**Figure 3:**
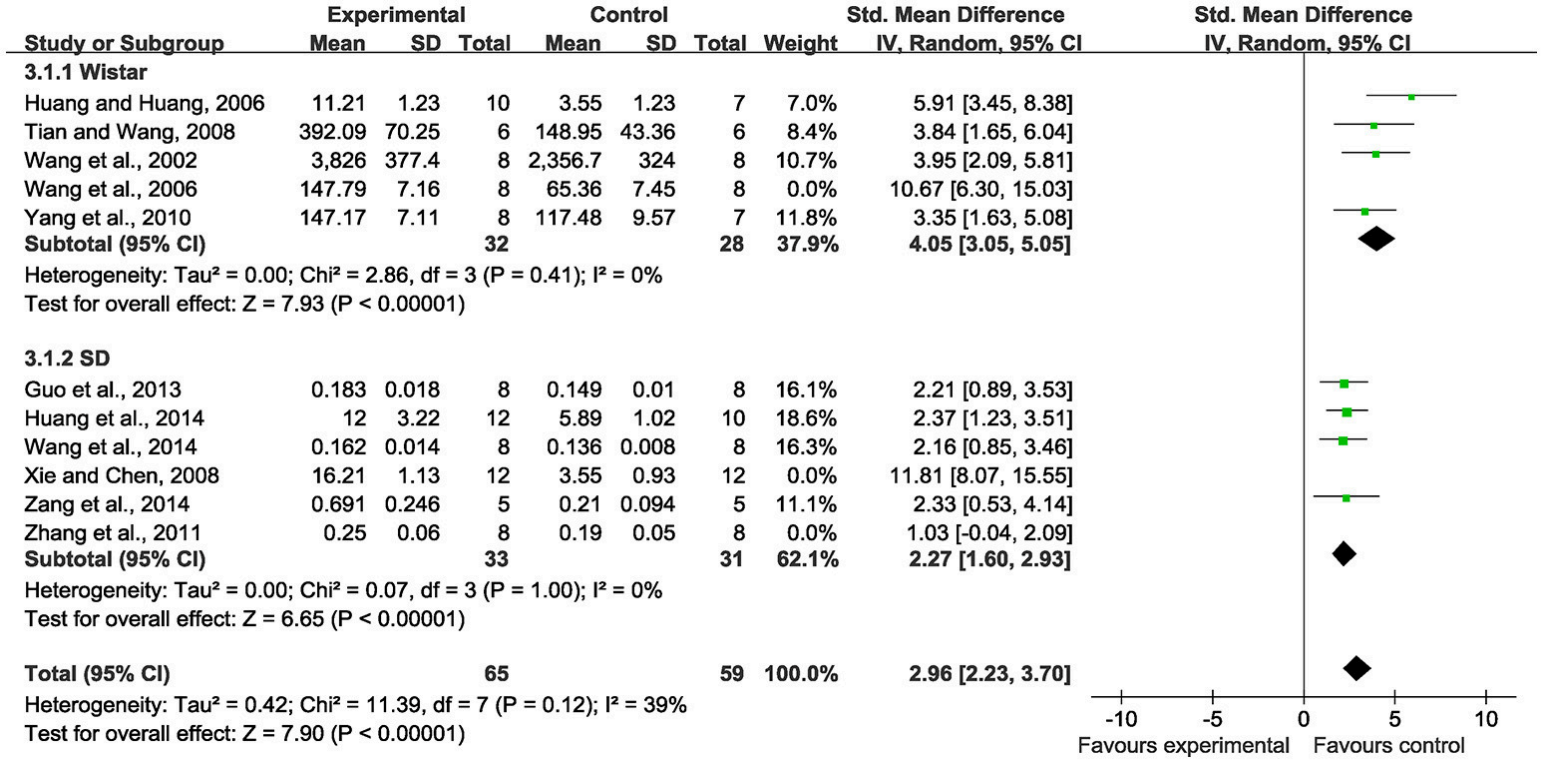
The forest plot: effects of Shexiang Baoxin Pills for increasing vascular endothelial growth factor protein expression.

**Figure 4:**
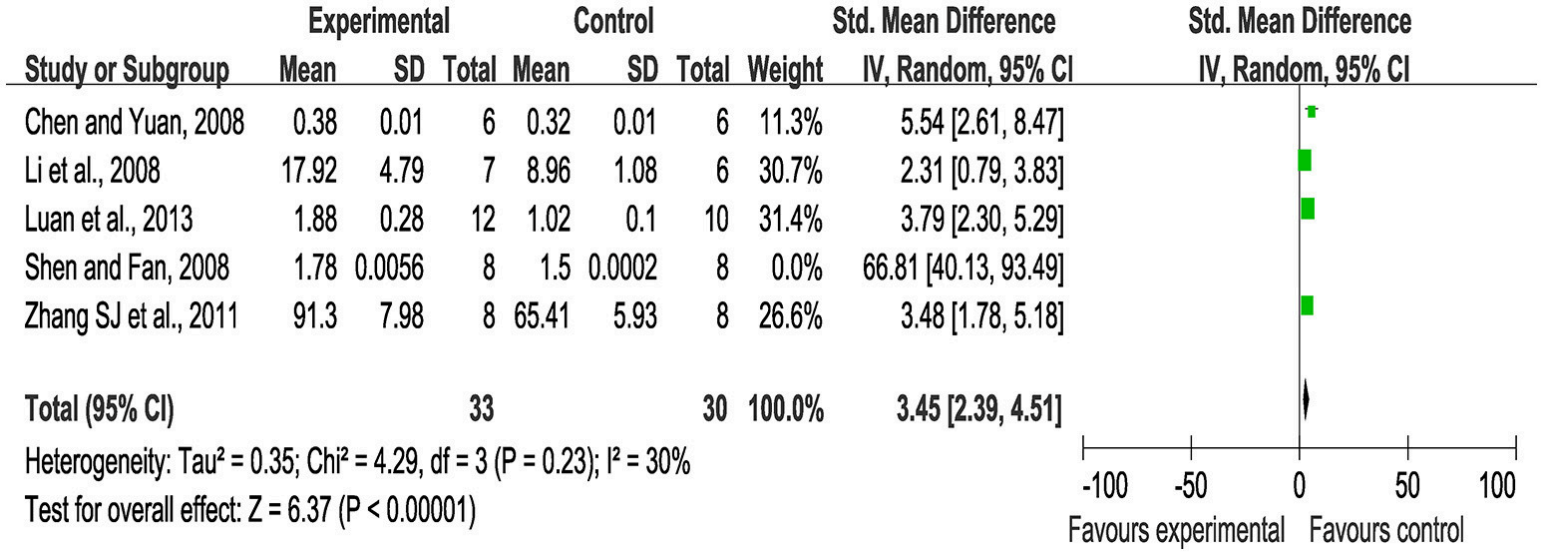
The forest plot: effects of Shexiang Baoxin Pills for increasing vascular endothelial growth factor mRNA expression.

Meta-analysis of 12 studies (Wang et al., 2002, 2006, 2014; Huang and Huang, 2006; Ling et al., 2007; Tian and Wang, 2008; Xie and Chen, 2008; Yang et al., 2010; Zhang Q. Y. et al., 2011; Guo et al., 2013; Huang et al., 2014; Zang et al., 2014) showed significant effects of SBP for increasing VEGF protein expression compared with the control (*n* = 193, SMD 3.54, 95% CI: 2.41~4.67, *P* < 0.00001; heterogeneity χ^2^ = 58.66, *P* < 0.00001, *I*^2^ = 81%). We conducted subgroup analysis according to the animal species. Wistar rats were used in five studies (Wang et al., 2002, 2006; Huang and Huang, 2006; Tian and Wang, 2008; Yang et al., 2010) as experimental subjects, among which there was obvious heterogeneity for the analysis (*n* = 76, SMD: 4.87, 95% CI: 3.15~6.60, *P* < 0.00001; heterogeneity χ^2^ = 11.24, *P* = 0.002, *I*^2^ = 64%). We performed a sensitivity analysis by sequentially excluding each individual study. After excluding the study (Wang et al., 2006), because the method of VEGF measure in this study was different from the others, meta-analysis of four studies (Wang et al., 2002; Huang and Huang, 2006; Tian and Wang, 2008; Yang et al., 2010) had more significant effect than the control for increasing VEGF protein expression (*n* = 60, SMD: 4.05, 95% CI: 3.05~5.05, *P* < 0.00001; heterogeneity χ^2^ = 2.86, *P* = 0.41, *I*^2^ = 0%), Figure 3. SD rats were used in six studies (Xie and Chen, 2008; Zhang Q. Y. et al., 2011; Guo et al., 2013; Huang et al., 2014; Wang et al., 2014; Zang et al., 2014). A sensitivity analysis was performed by sequentially excluding each study. After excluding the study (Xie and Chen, 2008) because of the same reason mentioned above and the study (Zhang Q. Y. et al., 2011) for the duration of the administration shorter than the other, meta-analysis of the other four studies showed the effect on increasing VEGF protein expression (*n* = 64, SMD: 2.27, 95% CI: 1.60~2.93, *P* < 0.00001; heterogeneity χ^2^ = 0.07, *P* = 1.00, *I*^2^ = 0%), Figure 3. There are five studies (Chen and Yuan, 2008; Li et al., 2008; Shen and Fan, 2008; Zhang S. J. et al., 2011; Luan et al., 2013) using VEGF mRNA as an outcome measure. Meta-analysis of these studies showed significant effects of SBP for VEGF mRNA expression than the controls (*n* = 79, SMD: 4.26, 95% CI: 1.68~6.83, *P* = 0.001; heterogeneity χ^2^ = 25.98, *P* < 0.00001, *I*^2^ = 85%). We used sensitivity analyses omitting one study at a time. One study (Shen and Fan, 2008) reported to make the embolic model with the obstructive glue. After excluding above study, meta-analysis of four studies (Chen and Yuan, 2008; Li et al., 2008; Zhang S. J. et al., 2011; Luan et al., 2013) showed significant effects on VEGF mRNA expression (*n* = 63, SMD: 3.45, 95% CI: 2.39~4.51, *P* < 0.00001; heterogeneity χ^2^ = 4.29, *P* = 0.23, *I*^2^ = 30%), Figure 4.

#### MVC

**Figure 5:**
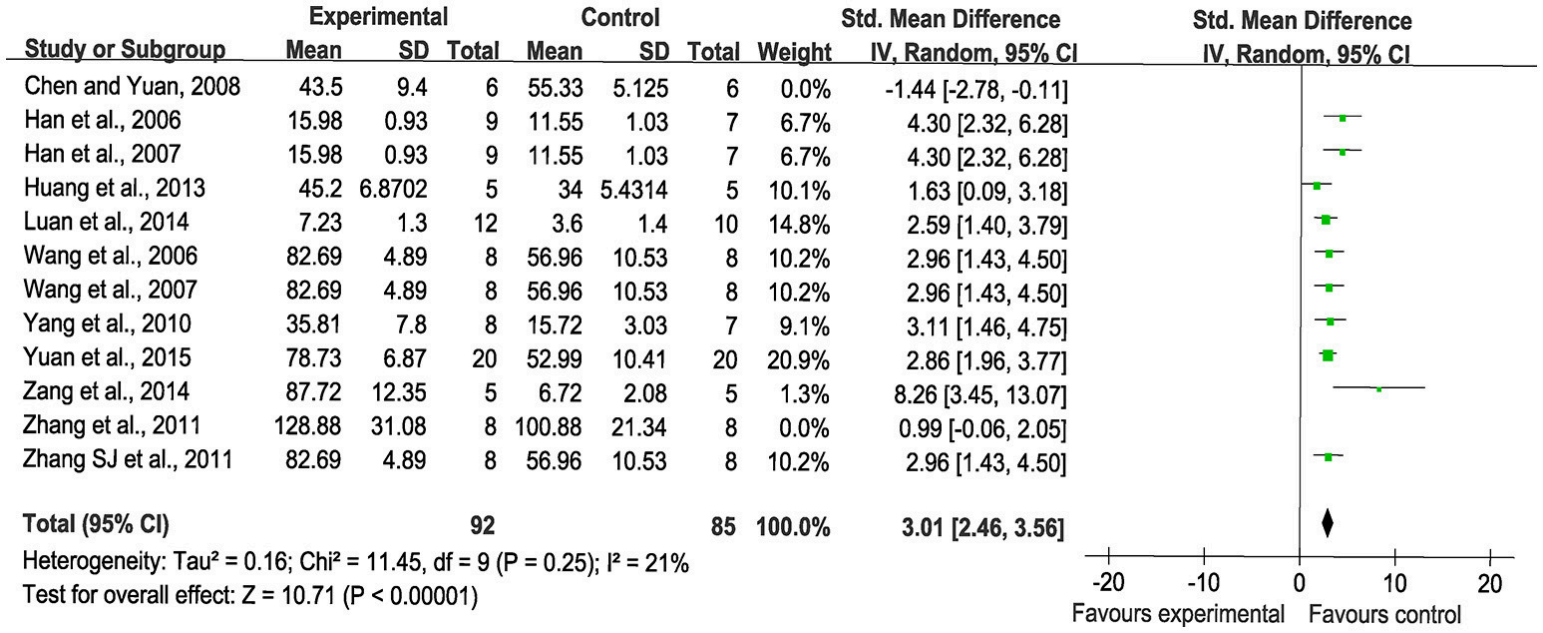
The forest plot: effects of Shexiang Baoxin Pills for increasing microvessel count.

Meta-analysis of twelve studies (Han et al., 2006, 2007; Wang et al., 2006, 2007; Chen and Yuan, 2008; Yang et al., 2010; Zhang Q. Y. et al., 2011; Zhang S. J. et al., 2011; Huang et al., 2013; Luan et al., 2014; Zang et al., 2014; Yuan et al., 2015) showed significant effects of SBP for increasing MVC compared with control (*n* = 205, SMD: 2.58, 95% CI:1.61~3.54, *P* < 0.00001; heterogeneity χ^2^ = 55.35, *P* < 0.00001, *I*^2^ = 80%). We used sensitivity analyses omitting one study at a time from the original analysis. The duration of the administration about two studies (Chen and Yuan, 2008; Zhang Q. Y. et al., 2011) is shorter than the other. The duration of the administration may lead to the heterogeneity. The studies above were considered as the potential sources of the heterogeneity. Meta-analysis of 10 studies (Han et al., 2006, 2007; Wang et al., 2006, 2007; Yang et al., 2010; Zhang S. J. et al., 2011; Huang et al., 2013; Luan et al., 2014; Zang et al., 2014; Yuan et al., 2015) indicated that SBP significantly improved MVC compared with the control (*n* = 177, SMD: 3.01, 95% CI: 2.46~3.56, *P* < 0.00001; heterogeneity χ^2^ = 11.45, *P* = 0.25, *I*^2^ = 21%), Figure 5.

## Discussion

### Summary of preclinical evidence

Twenty-five studies with 439 animals were included in the present study. The findings preliminarily demonstrated that SBP can reduce myocardial infarction area through an increase of VEGF and MVC in CHD, suggesting that SBP exerted potential cardioprotection largely through promoting angiogenesis.

### Strength and limitations

The strengths of the study are that systematic reviews of pre-clinical animal data could inform the planning to focus on the method of randomization to treatment group, blinded assessment of outcome, allocation concealment, and explicit sample size calculation (MacLeod et al., 2004), and to affirm the optimum dosage, duration and initial administration time identified for further “basic” research. Moreover, they could preclude unnecessary study replication, and contribute to both “reduction” and “refinement” in animal experimentation, improve the likelihood of success of future clinical trials (Murphy and Murphy, 2010). In the present study, we provided a novel study method for synthesizing animal studies that can independently evaluate the current preclinical evidence and possible mechanism of SBP for CHD.

Some limitations of the study are listed as follows: previous meta-analyses have suggested that animal studies, less rigorously designed, may overestimate treatment effects (Rooke et al., 2011). In the present study, all the studies failed to mention the blinded assessment of behavioral outcome, which made the overall quality of the containing studies moderate. It may lead to performance bias and detection bias (Kahan et al., 2014). Thus, the results should be interpreted with caution. Secondly, possible heterogeneity problems are as follows: different conditions include different animal species, dosage, duration, initial administration time, or administration route and various assessment methods of treatment effects, including the myocardial infarction area, VEGF, and MVC. Third, the patients suffered from CHD often with comorbidity. However, in the present study animal models used were healthy that were inconsistent with humans. Fourth, our search strategy included only Chinese and English databases leading to selective bias to some certain degrees (Guyatt et al., 2011). Finally, the primary aim of the studies reviewed was not to assess the angiogenesis effects of CHD. Some studies did not include an impartial measurement of myocardial infarction area, VEGF and/or MVC which may lead to bias in their outcome, or did not state the method of measurement.

### The forest plot and the sensitivity analysis

A forest plot is a graphical representation of the individual results of each study included in a meta-analysis together with the combined meta-analysis result according to the Cochrane Collaboration's definition (2014). The forest plot is able to demonstrate the degree to which data from multiple studies observing the same effect overlap with one another, allowing readers to see the heterogeneity among the results of the studies. Thus, the forest plot provides a quick visual representation of overall effect estimates and the heterogeneity (Callcut and Branson, 2009; Israel and Richter, 2011). The sensitivity analysis is based on Cochrane Reviews which is used to guide systematic reviews of intervention (Verhagen and Ferreira, 2014). A sensitivity analysis is a repeat of the primary analysis or meta-analysis, substituting alternative decisions or ranges of values for decisions that were arbitrary or unclear. In the present study, first of all, timing of initiation of treatment contributed to the heterogeneity according to myocardial infarction area outcome measure. Xie's study (Xie and Chen, 2008) administrated SBP after 1 week the models established other than after 1 day in the remaining seven studies (Wang et al., 2002, 2004; Li et al., 2008; Tian and Wang, 2008; Wang and Fan, 2008; Huang et al., 2013, 2014). Second, based on the VEGF outcome measure, a test method of the VEGF expression is undefinite in the study by Wang et al. (2006) while the remaining four studies (Wang et al., 2002; Huang and Huang, 2006; Tian and Wang, 2008; Yang et al., 2010) are clearly provided it; the duration of the administration by the study of Zhang Q. Y. et al. (2011) is shorter than that of the other five studies (Zhang Q. Y. et al., 2011; Guo et al., 2013; Huang et al., 2014; Wang et al., 2014; Zang et al., 2014); Shen et al. (Shen and Fan, 2008) used the embolic model with the obstructive glue while the other four studies (Chen and Yuan, 2008; Li et al., 2008; Zhang S. J. et al., 2011; Luan et al., 2013) used the ligate. Third, the duration of the administration by two studies (Chen and Yuan, 2008; Zhang Q. Y. et al., 2011) is shorter than the other 10 studies (Han et al., 2006, 2007; Wang et al., 2006, 2007; Yang et al., 2010; Zhang S. J. et al., 2011; Huang et al., 2013; Luan et al., 2014; Zang et al., 2014; Yuan et al., 2015) according to MVC outcome measure.

### Implications

Previous studies (Murphy and Murphy, 2010; Wei et al., 2013) suggested that the quality of the research design is an important factor affecting the outcome. A lower-quality study trends toward better outcomes, leading to the global estimated effect overstated (García-Bonilla et al., 2012). In the present study, the quality need be promoted by means of incorporating the ARRIVE guidelines (Kilkenny et al., 2010). In particular, we should focus on the method of randomization to treatment group, blinded assessment of outcome, allocation concealment and explicit sample size calculation (MacLeod et al., 2004). Second, according to the effect size, this study indicated that a comparison among varieties of the administration duration showed the shorter administration duration using SBP was less effectiveness in MVC improvement. The young animals distinguish from the pathology of CHD, a slowly developing and chronic disease, and with frequently co-morbidities such as diabetes, hypertension, atherosclerosis, or advanced age (Gianaros and Sheu, 2009; Mohamed Omer et al., 2016). Therefore, selecting suitable timing of initiation of treatment, the duration of the administration, the optimal animal models and a standardized test of VEGF expression are required in the future.

Ischemia and hypoxia of the myocardium induced by acute or chronic CHD reduces myocardial infarction area and promotes the angiogenesis by up-regulating VEGF (Ramakrishnan et al., 2014; Möbius-Winkler et al., 2016). Additionally, ischemic myocardium initiates a severe inflammatory response indirectly to promote the angiogenesis (Braunwald, 2013). The blood flow is interrupted so that the myocardium lacks the supply of the oxygen and the nutrition. Hypoxia induces inflammation, wherein inflammation causes hypoxia (Braunwald, 2013). Hypoxia inducible factor (HIF) is an essential factor induced in hypoxia condition which transactivates or transcriptionally regulates many hypoxia responsive genes such as VEGF (Leung et al., 1989). Then VEGF sites on endothelial cells as a stimulatory factor for proliferation, sprouting, migration, and luminal formation (Des Guetz et al., 2006; Konopka et al., 2013) and inhibits the calcium sensitive receptor to the apoptosis on myocardial ischemia from reperfusion injury (Hoffmann et al., 2013). Nevertheless, VEGF improves new blood vessels and enhances collateral development termed angiogenesis to guarantee the microcirculation establishment for the requirement of the ischemic myocardium (Banai et al., 1994; Tammali et al., 2011). In this study, SBP could reduce myocardial infarction area, up-regulate VEGF in the edge of myocardial infarction (Zhu et al., 2010), and increase blood vessel density, which promotes blood circulation to protect ischemic myocardium (Xiang et al., 2012). In addition, animal experiments have contributed to our understanding of angiogenesis for CHD by the signal way of VEGF or other growth factors (Hackam and Redelmeier, 2006; Hackam, 2007). Further investigation is needed to determine the specific signal way of angiogenesis. And the underlying molecular mechanisms also require further study.

Multiple active compounds in essence combined to enhance the effectiveness of TCM therapy (Zhou and Wang, 2014). SBP, known as the polypill, can be adapted for secondary prevention of cardiovascular disease based on existing evidence confirming that SBP can reduce the ischemia myocardium to keep the myocardial function and promote the angiogenesis to increase the blood flow (Lafeber et al., 2013; Working Group on the Summit on Combination Therapy for CVD et al., 2014). SBP also plays an important role in anti-thrombosis and anti-artherosclerosis (Zhou and Wang, 2014). SBP for the second prevention of CHD need to be further confirmed by RCTs.

## Conclusion

The present study provided the preliminary preclinical evidence that SBP can reduce myocardial infarction area, largely through promoting angiogenesis. This study paves a new way to elucidate the angiogenesis of CHD through exploring the function of SBP at marginal zone of infarcted myocardium.

## Author contributions

KZ, JZ, XB, QZ, GZ, and YW designed the study; KZ and JZ collected the data; KZ and JZ performed all analyses; KZ, GZ, and YW wrote the manuscript. All authors contributed to writing of this manuscript.

### Conflict of interest statement

The authors declare that the research was conducted in the absence of any commercial or financial relationships that could be construed as a potential conflict of interest. The reviewers OC and LX and handling Editor declared their shared affiliation, and the handling Editor states that the process nevertheless met the standards of a fair and objective review.
